# Extracellular vesicles shape tumourigenesis: from signalling networks to systemic progression

**DOI:** 10.1007/s10555-026-10377-4

**Published:** 2026-09-23

**Authors:** Stephanie B. Telerman, Janusz Rak, Adam Telerman

**Affiliations:** 1 CRUK Cambridge Institute, University of Cambridge, Li Ka Shing Centre, Cambridge, UK; 2 McGill University, The Research Institute of the McGill University Health Centre, Montreal, QC Canada; 3 Institut Gustave Roussy, Villejuif, France

**Keywords:** TSAP6/STEAP3, TCTP, DDX3, Small extracellular vesicles, Tumour reprogramming/reversion, Intercellular communication

## Abstract

Extracellular vesicles (EVs) are established mediators of long-range intercellular communication in cancer, transferring oncogenic and regulatory cargo that reshapes recipient cells across tissues. Recent integrative multi-omics analyses of small EVs (sEVs) from healthy human donors consistently identify TSAP6/STEAP3 among the most abundant and reproducible sEV components, providing a physiological context for its role in sEV biology. Rather than acting solely as a downstream effector of p53, TSAP6 functions within a broader regulatory network by associating with TPT1/TCTP, which engages DDX3 and promotes the incorporation of RNAs, including microRNAs, into sEVs. The resulting p53–TSAP6–TCTP–DDX3 axis constitutes a wide-reaching regulatory system with non-cell-autonomous impact on cellular communication. Notably, TCTP sustains sEV-mediated signalling even in p53-mutant contexts. We suggest that these findings are consistent with other advances in cancer linking oncogenic transformation with EV biology. Together, these observations support regulated EV-mediated information transfer as a mechanism contributing to cancer progression and systemic cellular reprogramming. Conversely, reduction in EV abundance, altered cargo content, or signalling capacity may contribute to the activation of the tumour reversion programme in TCTP-dependent experimental models.

## Introduction

The rapid expansion of EV research has produced a rich body of work. Here, we focus on recent discoveries [1–3] and their integration with previous studies [4, 5], which together point to an emerging conceptual consolidation in the EV field. Two directions are particularly relevant to the framework considered here: first, the role of EVs in non-cell-autonomous oncogenic signalling [2, 3], often referred to as the horizontal transfer of oncogenic material, and second, the contribution of tumour-derived EVs to systemic cancer-associated pathologies [6, 7].

The term extracellular vesicles (EVs) is an umbrella term encompassing lipid bilayer-delimited vesicles released by cells and capable of carrying a wide range of molecules, including signalling receptors, membrane-bound ligands, adhesion molecules, enzymes, lipids, nucleic acids and metabolites [8, 9]. EVs are heterogeneous in size, biogenesis, molecular composition, and function [10, 11], reflecting the involvement of distinct trafficking effectors and vesicle formation machineries [11]. Advances in EV isolation and characterisation technologies have been central to resolving this diversity and defining distinct EV populations [12]. Among these, small extracellular vesicles (sEVs), often within the 30–150 nm range, can originate through different biogenetic pathways [13]. Exosomes constitute the endosome-derived subset of sEVs, originating as intraluminal vesicles within multivesicular endosomes and released upon fusion with the plasma membrane [14, 15]. Because this endosomal origin is often difficult to establish, particularly *in vivo*, the broader term sEV is used when the precise biogenetic origin is uncertain [15].

EV-mediated communication can operate locally through autocrine and paracrine signalling or at a distance following dissemination through the circulation [10, 11]. This capacity for local and long-range molecular exchange is particularly relevant to cancer as a multicellular and systemic disease. A central question is therefore how the molecular pathways governing sEV biogenesis, composition and biological activity intersect with oncogenic transformation and contribute to reprogramming of the tumour microenvironment and systemic dissemination of disease. It is important to determine whether these pathways influence the magnitude and biological consequences of EV-mediated communication and represent potential therapeutic targets.

In the following sections, we integrate these emerging findings within the framework of the Tumor Suppressor Activated Pathway-6 (TSAP6/STEAP3) and Translationally Controlled Tumor Protein (TCTP/TPT1) axis, which lies at the interface of stress biology, vesicle biogenesis, EV-mediated signalling, and the balance between malignant progression and tumour reversion [1, 2].

## The p53-TSAP6/STEAP3-TCTP axis

A recent multi-omics analysis of circulating human sEVs provides physiological context for vesicle-associated pathways in humans [1]. Using high-resolution iodixanol density gradients to isolate sEVs, combined with quantitative proteomics and lipidomics, this study separated vesicles from abundant plasma protein and lipoprotein contaminants and identified a conserved “core” signature of 182 proteins and 52 lipids shared across individuals. Importantly, TSAP6/STEAP3 was detected within this conserved feature set, alongside established endosomal and trafficking proteins such as flotillins, Rabs, annexins, and SNARE-related factors. These findings establish the presence of TSAP6/STEAP3 in circulating sEVs from healthy individuals, although the cellular origin and physiological state of the cells releasing these vesicles remain unknown [1].

**Fig. 1:**
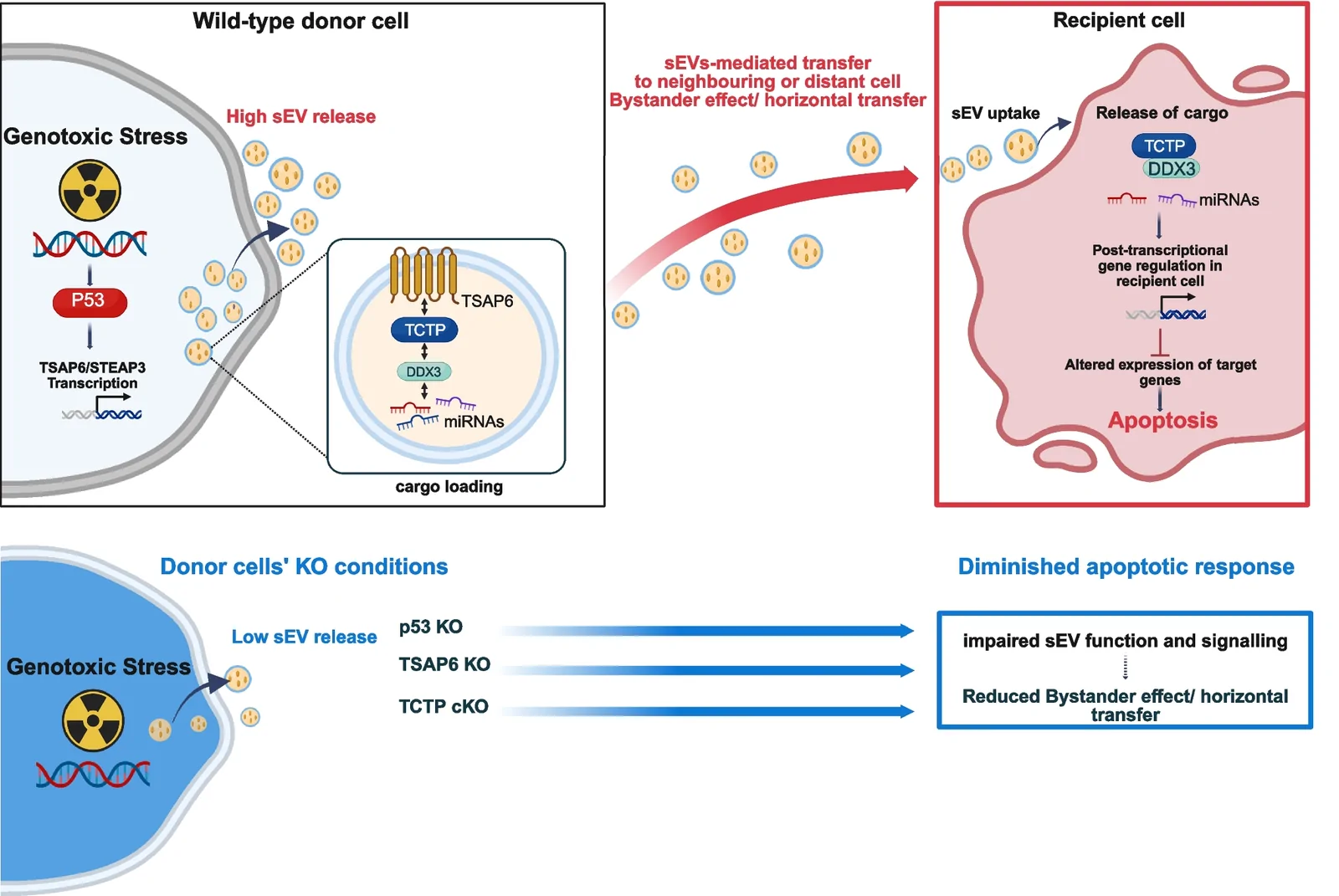
The p53–TSAP6/STEAP3–TCTP–DDX3–microRNA pathway in stress-induced sEV signalling. Upper panel: Following genotoxic stress, p53 induces TSAP6/STEAP3 expression and promotes the release of sEVs. TSAP6/STEAP3 interacts with TCTP and facilitates its vesicular export. TCTP regulates sEV release and cargo composition and interacts with DDX3, which promotes the recruitment of RNAs, including microRNAs, into sEVs. In recipient cells, transferred vesicular cargo contributes to the apoptotic bystander response (red arrow). Lower panel: Loss of p53 or TSAP6/STEAP3, or conditional deletion of TCTP, disrupts this pathway at distinct levels, leading to reduced sEV release and/or altered cargo composition. Consequently, sEV-mediated bystander signalling is attenuated (blue arrows). The figure was created with BioRender.com

TSAP6 (Fig. 1) was first identified in a differential cDNA display screen designed to expand the molecular landscape of p53-induced apoptosis beyond classical cell-cycle regulators [16]. Using the temperature-sensitive LTR6 system, restoration of wild-type p53 activity revealed ten previously uncharacterised transcripts, among which TSAP6 was selected for further analysis.

Subsequent work demonstrated that TSAP6 is a direct transcriptional target of p53 through a specific p53 response element in its promoter [17]. TSAP6 was reported to modulate apoptosis and cell-cycle progression through interactions with Nix/BNIP3L and Myt1 kinase, linking it to pro-apoptotic signalling and G2/M checkpoint control. A significant advance came from evidence that TSAP6 regulates nonclassical protein secretion. TSAP6 directly binds TCTP (reviewed in more detail in the next section) and its overexpression increases extracellular TCTP release. This secretion was insensitive to inhibition of the conventional ER–Golgi pathway, and TCTP was detected in small vesicle fractions consistent with exosomes [18], linking the p53-responsive TSAP6 pathway to regulated vesicle-mediated protein export.

This concept was further consolidated by Arnold Levine and colleagues, who demonstrated that p53 activation induces exosome secretion as part of the DNA damage response [19]. In lung tumour cell lines, γ-irradiation increased exosome production in a p53-dependent manner, and this effect was mechanistically linked to TSAP6 induction. TSAP6 expression alone was sufficient to enhance exosome release.

The biochemical identity of TSAP6 was further clarified following its characterisation as STEAP3, a member of the Six-Transmembrane Epithelial Antigen of Prostate (STEAP) family [20]. Ohgami and colleagues showed that STEAP3 colocalises with the transferrin-cycle endosome and functions as an endosomal ferrireductase required for efficient transferrin-dependent iron uptake in erythroid cells; Steap3-deficient mice consequently develop microcytic, hypochromic anaemia [20]. Consistent with the identity of TSAP6 and STEAP3, TSAP6-null mice generated by Lespagnol and colleagues showed abnormal reticulocyte maturation and defective transferrin receptor downregulation [4]. TSAP6 was also detected in trans-Golgi and endosomal-vesicular compartments, with partial colocalisation with transferrin receptor and early endosomal markers, while primary TSAP6-null cells exhibited markedly reduced exosome release following genotoxic stress [4].

The two studies describe complementary aspects of the same TSAP6/STEAP3 gene product, rather than separate molecular pathways in erythroid cells. Pan and Johnstone had previously shown that transferrin receptor is selectively externalised in vesicles during reticulocyte maturation [14]. Together, these observations provide genetic and cellular evidence linking TSAP6/STEAP3 to vesicular trafficking during reticulocyte maturation and to regulated exosome secretion [4]. Human genetics further confirmed the physiological relevance of STEAP3, with a nonsense mutation in TSAP6/STEAP3 identified in congenital hypochromic anaemia [21].

PanCancer analysis (https://www.cbioportal.org/) indicates that STEAP3 is infrequently altered at the genomic level in human cancers, with no recurrent mutational hotspots and only uncommon high-level copy-number or structural alterations, although STEAP3 overexpression has been described in diverse tumour types.

Taken together, these findings establish a framework in which the initial identification of TSAP6 through differential cDNA screening [16], its regulation by p53 [17], its role in vesicular trafficking [18], its involvement in p53-dependent exosome regulation [19], and evidence from genetic ablation models [4] collectively link the p53-TSAP6-TCTP axis to extracellular vesicle biology. Within this context, TSAP6 functions as a membrane protein involved in the regulation of p53-dependent vesicle-mediated intercellular communication.

A logical next step is to determine in large patient cohorts whether TP53 status, including specific mutant classes, correlates with TSAP6 expression, vesicle abundance or EV cargo composition, and whether TSAP6-containing sEVs have biomarker or functional relevance in tumour progression. Within the physiological framework provided by circulating human sEVs [1], STEAP3 was detected in all 38 plasma EV proteomes, whereas TCTP was detected in 18 out of 38. Because these measurements were obtained from bulk sEV preparations, they do not establish whether STEAP3 and TCTP coexist within the same vesicles or are partitioned among distinct sEV subpopulations (https://evmap.shinyapps.io/evmap). Conversely, the detection of both proteins in circulating sEV preparations indicates that their presence in sEVs is not restricted to genotoxic stress or cancer. Furthermore, evidence indicates that the functions of TCTP in sEVs extend beyond the canonical p53–TSAP6 axis [2], as discussed in the following section.

## Decoupling TCTP vesicular signalling from the p53 axis

Although TCTP biology has been extensively reviewed [22], we briefly outline it here to contextualise its role in EV secretion and cargo regulation. The translational control of the protein later recognised as TCTP was already evident in early studies by George Brawerman and colleagues [23]. TCTP was subsequently identified by Susan MacDonald and colleagues as histamine-releasing factor (HRF) [24], establishing an extracellular role in allergic inflammation and foreshadowing its broader function in intercellular signalling. In our studies of the tumour reversion programme, we used differential gene expression approaches, including Megasort and Massively Parallel Signature Sequencing (MPSS), and detected 248 signals corresponding to TCTP mRNA in U937 tumour cells, compared with only 2 in the revertant US cells [25]. Furthermore, inhibition of TCTP also promoted the reorganisation of breast cancer cells into ductal-like structures [25, 26].

TCTP is involved in growth control, cytoskeletal organisation, protein synthesis and the elongation machinery [22]. Further insight into TCTP function came from studies by Edouard Azzam and colleagues in human fibroblasts and osteosarcoma cells, which identified TCTP as a component of the mammalian DNA damage response [27].

Independently, a series of genetic studies by Kwang-Wook Choi and colleagues in *Drosophila* established Tctp as a regulator of genome stability and chromatin organisation [28, 29].

The anti-apoptotic activity of TCTP is among its best-characterised functions [25, 30, 31]. Biological and structural studies showed that TCTP contains a BH3-like domain, providing a mechanistic basis for its interaction with BCL2-family regulators of apoptosis [32]. TCTP also exhibits the unusual ability to activate the anti-apoptotic function of the BCL2-family member Bcl-xL [32].

**Fig. 2:**
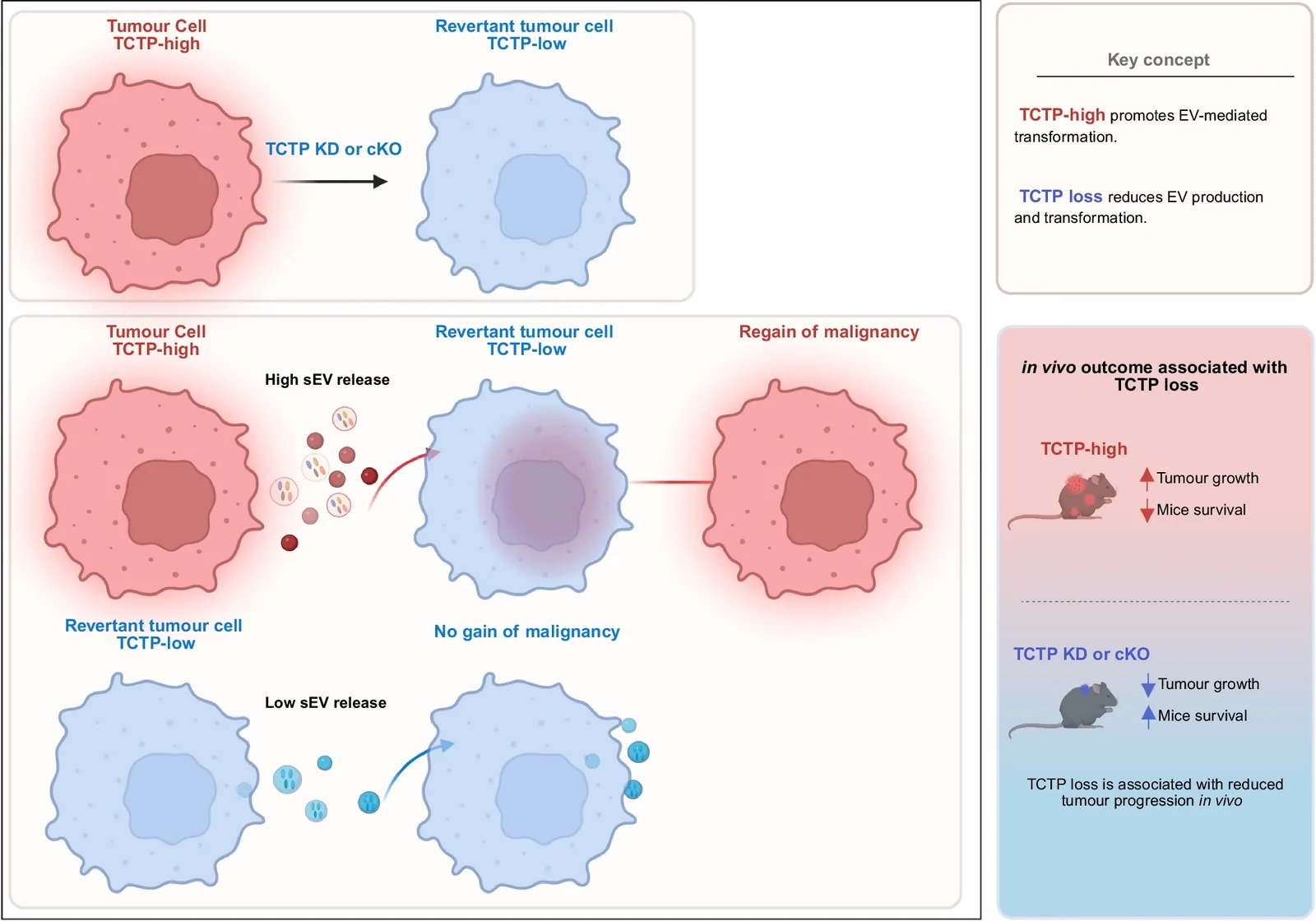
TCTP-dependent sEV communication regulates malignant growth across several biological systems, and TCTP knockdown or conditional knockout induces tumour reversion. Left panel: TCTP-high tumour cells (red) include the human breast cancer cell lines MCF-7, T-47D, SK-BR-3 and MDA-MB-231, as well as the murine ITR-1 tumour cell line. TCTP knockdown (KD; blue) in breast cancer cells or conditional *Tctp* knockout (cKO; blue) in ITR-1 cells reduces malignant growth, generating a TCTP-low revertant phenotype (blue). sEVs released by TCTP-high parental tumour cells (red) restore the malignant phenotype of TCTP-low revertant cells (blue), whereas sEVs released by TCTP-low revertant cells (blue) do not. Right panel: TCTP-high status (red) is associated with greater tumour growth and shorter survival *in vivo*, whereas TCTP loss (blue) reduces tumour development and significantly prolongs survival in *Trp53*-null mice. The figure was created with BioRender.com

The discovery that TCTP is secreted via exosomes expanded its functional scope [18], although the consequences of vesicular release remained unclear. Recent work shows that TCTP contributes to sEV production, cargo composition and biological activity in recipient cells [2] (Figs. 1 and 2). These findings revealed an apparent paradox: although intracellular TCTP has a well-established anti-apoptotic function, it is required for sEVs released following genotoxic stress to transmit pro-apoptotic signals. This apparent paradox can be resolved by distinguishing the intracellular function of TCTP from its role in vesicular communication. In donor cells, TCTP contributes to sEV production and cargo selection, but does not itself determine whether the transmitted signal is pro- or anti-apoptotic. Rather, the response of recipient cells reflects the physiological state of the donor cell and the molecular cargo incorporated into the vesicles. Accordingly, sEVs released from irradiated cells undergoing genotoxic stress can transmit a pro-apoptotic signal (Fig. 1), whereas TCTP-dependent sEVs released from tumour cells can convey tumour-promoting activity (Fig. 2) [2].

The pro-apoptotic response transmitted by sEVs from irradiated cells is consistent with the radiation-induced bystander effect established by Azzam, Little and colleagues [33]. In inducible Tctp-knockout thymocytes, the sEV-mediated component of this response was TCTP-dependent, with Tctp deletion reducing sEV release and altering vesicular protein and RNA content [2]. The accompanying changes in RNA cargo provided insight into the underlying mechanism. RNAs, including mRNAs and miRNAs, are established functional components of vesicular cargo that can be transferred to recipient cells [8].

Under the experimental conditions tested, no direct binding of TCTP to RNA was detected. Instead, TCTP interacted directly with the RNA helicase DDX3 and enhanced the association of DDX3 with let-7c-5p [2]*.* Comparison with miRTarBase showed that 43 of 128 miRNAs annotated as targeting DDX3X were also identified in the anti-TCTP CLIP dataset [2, 34]. Together, these findings support a model in which TCTP contributes to miRNA recruitment into sEVs through DDX3.

The role of TCTP in sEV biology is not limited to stress-induced secretion. In tumour-prone *Trp53*-null mice, conditional deletion of *Tctp* reduced tumour formation and prolonged survival in the *Trp53*–/–;*Tctp*–/f– double-knockout model [2]. In a subset of mice, further induction of *Tctp* deletion resulted in regression of established tumours, suggesting that TCTP contributes to both tumour development and maintenance in the absence of p53 (Fig. 2).

Because circulating tumour-derived sEVs were not directly measured in these mice, their potential contribution was examined using Inducible Tumour Reversion-1 (ITR-1) cells, derived from a sarcoma arising in a *Trp53*–/–;*Tctp*–/f mouse. Conditional deletion of the remaining *Tctp* allele (Fig. 2), generating *Trp53*–/–;*Tctp*–/f– cells, impaired growth, reduced sEV secretion and decreased the protein and RNA content of the remaining vesicles. Supplementation (Fig. 2) with sEVs from TCTP-proficient parental ITR-1 cells restored growth, supporting a TCTP-dependent, vesicle-mediated tumour-promoting circuit [2].

A similar dependence on TCTP was observed in human breast cancer cells. TCTP knockdown reduced spontaneous sEV secretion in four cell lines with distinct *TP53* status: MCF-7 (wild-type *TP53*), T-47D (L194F), SK-BR-3 (R175H), and MDA-MB-231 (R280K) [2]. TCTP depletion also impaired growth, which could be restored by supplementation with sEVs isolated from the corresponding TCTP-proficient parental cells (Fig. 2). The consistency of these effects across wild-type and mutant *TP53* backgrounds, including the well-characterised gain-of-function p53 mutants R175H and R280K, indicates that TCTP-dependent sEV secretion and growth-supporting activity are maintained across distinct p53 contexts in these models [2]. Whether this also applies to human tumours remains to be established. Because these analyses were performed on bulk sEV preparations, they do not establish whether TCTP depletion affects all released vesicles similarly or changes the relative abundance and composition of distinct sEV populations.

The dependence of these tumour models on TCTP also identifies a potential therapeutic vulnerability. Treatment with sertraline, which binds TCTP and inhibits its function, prolonged survival in *Trp53*–/– mice, suggesting that TCTP may remain therapeutically targetable in the absence of p53 [2].

The persistence of TCTP-dependent sEV secretion and signalling when p53 is mutated or absent supports a functional decoupling of this vesicular activity from the canonical p53 stress-response axis in the models examined. Nevertheless, TCTP and p53 remain connected through a reciprocal regulatory loop: TCTP promotes MDM2-dependent p53 degradation, whereas p53 directly represses TCTP transcription [35]. Loss of p53 wild type function may therefore relieve transcriptional restraint on TCTP, while elevated TCTP may further reduce p53 levels. In the same study, high TCTP expression in a cohort of 508 breast cancers was associated with poorly differentiated, aggressive tumours and independently predicted poorer disease-free survival, whether considering distant events or all events [35].

Whether TCTP-dependent sEV signalling links the reciprocal p53–TCTP loop to tumour aggressiveness, patient outcome and systemic consequences remains to be established in human tumours. More broadly, dysregulated TCTP activity and p53 dysfunction may jointly reshape intracellular programmes and extracellular communication, thereby contributing to tumour progression.

## EVs as vectors of oncogenic transfer and systemic cancer progression

Over the past two decades, a series of foundational observations have established that EVs are not only regulated by oncogenic transformation but can also carry functional oncogenic material [5, 36, 37]. Two related dimensions are considered here: first, the horizontal transfer of oncogenic material between tumour cells and surrounding cellular populations; and second, EV-mediated communication at distant sites, including its contribution to systemic cancer-associated responses and metastatic dissemination.

We provided the first evidence that glioma cells expressing the mutant oncogenic receptor EGFRvIII release EVs enriched in this oncoprotein. These cancer-derived EVs can fuse with EGFRvIII-negative cells and activate MAPK and Akt signalling, thereby promoting proliferation, survival and angiogenesis [5]. This study established the principle of horizontal oncogenic transfer through tumour-derived vesicles, whereby biologically active oncogenic material released by one tumour-cell population can be acquired by another and modify signalling and phenotype in the recipient cells. More recently, EVs from mesenchymal glioma stem cells were shown to deliver EGFR to endothelial cells and promote a VEGF-independent form of tumour neovascularisation, termed vasectasia, that is resistant to conventional anti-angiogenic agents [3]. EGFR-carrying cancer EVs can also interact with microglial cells and alter their molecular properties [38]. Thus, EV-mediated oncogenic transfer can operate not only between tumour-cell populations but also across different cellular compartments of the tumour microenvironment.

This principle is not restricted to EGFRvIII. For example, EV-mediated transfer of tumour-associated oncogenic material has also been reported for amplified wild-type EGFR [39], MET [40], and oncogenic H-RAS DNA [41], among others.

These studies encompass the transfer of both protein and nucleic acid cargo and collectively demonstrate that EVs can transfer biologically active tumour-associated molecules between cells. Importantly, the resulting signalling or phenotypic effects do not necessarily imply stable oncogenic transformation. Normal recipient cells can impose tumour-suppressive barriers on horizontal transformation [42].

A related but distinct question is how oncogenic signalling regulates the production and composition of the EVs involved in these interactions. Following ligand stimulation, EGFR trafficking into endosomes and intraluminal vesicles is regulated by receptor activation, ubiquitination and ESCRT-dependent sorting [43], while oncogenic variants such as EGFRvIII differ from ligand-activated wild-type EGFR in their trafficking properties [44].

EGFR trafficking can also be altered under cellular stress [43]. In glioma cells, pharmacological inhibition of neutral sphingomyelinase activity with GW4869 reduces EGFR incorporation into EVs [38]. Analyses at the single-EV level further showed that glioblastoma cells with high and uniform EGFR expression release heterogeneous EV populations, only a fraction of which contains detectable EGFR [38].

Other oncogenic pathways also influence EV production, although their effects vary in magnitude and mechanism. In breast epithelial cells, enforced expression of different oncogenes had distinct effects on EV release and composition. MYC and AURKB elicited particularly high levels of EV release, with MYC-dependent vesiculation strongly linked to ceramide metabolism and AURKB-dependent release showing a strong dependence on the ESCRT machinery [45]. In the same study, individual oncogenes selectively altered EV protein composition, and an inverse relationship between MYC upregulation and RAS/MEK/ERK pathway activation was observed in the regulation of EV release in some tumour cells, further illustrating that different oncogenic drivers can differentially influence EV production and cargo [45].

EV-mediated communication within the tumour microenvironment extends beyond the transfer of oncogenic drivers themselves. EV exchange between distinct cancer cell populations [46], or uptake by non-transformed recipients [47], may modulate tumour phenotypes without stable transfer of a canonical oncogenic driver. Communication also occurs in the opposite direction, from stromal cells to cancer cells. Endothelial cell-derived EVs [48] and astrocyte-derived EVs [49] can modify tumour-cell signalling; notably, astrocytic EV-associated miR-19a downregulates PTEN in breast cancer cells metastasising to the brain [49]. Endothelial EVs can likewise induce mesenchymal changes in proneural glioma cells [48]. Fibroblast-secreted exosomes can promote breast cancer-cell motility through Wnt–PCP signalling; trafficking within recipient cancer cells promotes the association of tumour-cell-derived Wnt11 with these fibroblast-derived exosomes [50].

Collectively, these findings establish EVs as mediators of non-cell-autonomous oncogenic signalling and reciprocal communication between tumour and stromal compartments. Horizontal oncogenic transfer represents one component of this process: EVs can transmit functional oncogenic material between cells, while other EV-mediated signals can modify the behaviour of tumour and stromal populations and contribute to remodelling of the local tumour microenvironment.

Beyond these local and regional effects on tumour and stromal cells, EV-mediated communication also extends to the systemic level, where tumour-derived vesicles can influence distant organs and contribute to metastatic progression. Cancer is a systemic disease characterised by paraneoplastic alterations and metastatic dissemination [51], and tumour-derived EVs can contribute to these processes.

Seminal studies showed that melanoma-derived EVs condition bone marrow-derived cells and distant tissues to facilitate metastatic colonisation and contribute to the establishment of pre-metastatic niches [40]. Pancreatic cancer-derived exosomes provide a further example of extracellular matrix remodelling at a distant site: their uptake by Kupffer cells promotes TGF-β secretion and fibronectin production by hepatic stellate cells, contributing to the formation of the liver pre-metastatic niche [52]. Subsequent work linked specific exosomal integrins to organ-specific metastatic tropism, with α6β4 and α6β1 associated with lung metastasis and αvβ5 with liver metastasis [6]. More recently, systemic sEV-mediated communication was shown to involve host cells at distant sites: CXCL13-reprogrammed interstitial macrophages in the lung pro-thrombotic niche release integrin-β2-containing sEVs that promote thrombosis and metastatic progression [7]. Together, these studies illustrate how cancer-associated EV communication can remodel distant tissues and influence metastatic progression.

These effects are not uniformly pro-metastatic. Exosomes from poorly metastatic melanoma cells can restrict metastatic dissemination by promoting antitumour immune surveillance [53]. In a distinct setting, EV-associated genomic DNA can stimulate innate immune responses in the liver that limit metastatic colonization [54]. Thus, EVs can either promote or restrain metastasis, depending on their cargo and the cellular context of the recipient tissue.

Many mediators of these systemic responses, including chemokines, coagulation factors and integrins, are not themselves oncogenic drivers but act downstream of oncogenic transformation. Since oncogenic drivers regulate both the EV repertoire [36] and the expression of cancer-associated soluble mediators [55], oncogenic transformation can affect EV-mediated communication at multiple levels, from vesicle production and cargo composition to local intercellular transfer and systemic responses. These observations link EV biogenesis and cargo regulation to oncogenic signalling and cellular stress responses.

As discussed above in Sect. 3, TCTP provides an experimentally defined link between sEV signalling and tumour-associated growth in models of horizontal transfer. By regulating vesicle secretion and cargo content, TCTP modulates sEV-mediated tumour-promoting communication, with restoration of TCTP-proficient sEVs rescuing the growth defect associated with TCTP depletion [2] (Fig. 2). Together, these findings identify TCTP as a regulator of sEV biology and place TCTP-dependent sEV signalling within the broader framework of EV-mediated communication in cancer.

## Knowledge gaps and future directions

One question that remains unresolved, and emerges from the preceding sections, is whether EV heterogeneity has functional consequences and, if so, how these differences shape biological responses. Bulk analyses describe the aggregate composition of EV preparations but cannot determine how cargo is distributed among individual vesicles. Analyses of individual EVs and extracellular particles have revealed heterogeneous distributions of selected cancer-associated molecules [56]. As illustrated by the glioblastoma models discussed above, glioblastoma cells uniformly expressing oncogenic EGFR produce sEV populations with or without detectable EGFR. The combined biological effects of these populations remain unclear, as do the respective pathways responsible for their generation within the molecular milieu of the parental cancer cells [38]. More broadly, it remains to be determined whether distinct EV populations target different recipient cells, act cooperatively or competitively, and how their effects intersect with soluble mediators and direct cell–cell or cell–matrix interactions.

The p53–TSAP6/STEAP3–TCTP pathway raises a related but distinct set of questions. TCTP depletion reduces spontaneous sEV release and decreases the protein and RNA content of the released vesicles in tumour models with different p53 backgrounds [2]. It remains unclear whether these quantitative and compositional changes affect all released vesicles similarly or reflect selective effects on particular EV populations, through which steps of vesicle biogenesis and cargo loading TCTP acts, and how distinct *TP53* alterations influence these processes in human tumours. Addressing these questions will require perturbation of candidate regulatory pathways together with analyses at the single-EV level and functional testing of defined EV populations. Current approaches can analyse selected markers or molecular signatures on individual vesicles, but linking these measurements to vesicle origin, cargo delivery and biological effect remains difficult [57, 58].

These unresolved questions also have implications for biomarker development. STEAP3 belongs to a conserved set of proteins identified in circulating plasma sEVs from healthy individuals, providing a physiological reference for studies of disease-associated changes [1]. Its presence in the normal circulating repertoire indicates that diagnostic information is unlikely to reside in STEAP3 detection alone but may depend on quantitative changes or on its association with other markers in defined EV populations. The ExoDx Prostate IntelliScore illustrates this combinatorial approach by integrating *PCA3*, *ERG*, and *SPDEF* RNA measurements in urinary exosomes to estimate the risk of high-grade prostate cancer at initial biopsy [59].

Therapeutically, two complementary strategies can be considered. Cell-derived EVs can be modified by manipulating donor cells or by loading isolated vesicles with therapeutic cargo. In a preclinical study, exosomes derived from fibroblast-like mesenchymal cells were engineered to carry siRNA or shRNA targeting *KRAS*^G12D and suppressed pancreatic cancer in mouse models [60]. Conversely, pathological EV-mediated communication might be weakened by interfering with vesicle biogenesis, cargo loading, release, binding or uptake [9, 61]. A key challenge is selectivity, as the same processes also support physiological intercellular communication. A priority is therefore to identify disease-relevant EV populations and their regulatory nodes, rather than to suppress EV-mediated communication indiscriminately. The effects observed with sertraline in the models discussed here provide a basis for testing whether pharmacological targeting of TCTP can alter tumour-relevant EV communication in human cancers [2].

The broader question is therefore how changes in EV production, composition and delivery are translated into biological responses. The outcome depends on the state of the donor cell, the vesicles released and the biological context of the recipient cell. The pathways examined in this review illustrate several experimentally defined routes through which intracellular stress and oncogenic signalling can influence EV production, cargo composition and intercellular information transfer, without implying that any single pathway represents a universal mechanism for EV biogenesis or function. Further progress will depend on establishing causal relationships between EV-regulatory pathways, EV cargo, and recipient-cell responses. From this perspective, tumour communication can be considered as a regulated interaction between donor cells, extracellular vesicles and recipient tissues, rather than as a property of the cancer cell alone.

## Data Availability

No datasets were generated or analysed during the current study.
